# The evolutionary extortion game of multiple groups in hypernetworks

**DOI:** 10.1038/s41598-022-25294-z

**Published:** 2022-12-05

**Authors:** Aizhong Shen, Zilin Gao, Xiang Gao, Dan Cui

**Affiliations:** 1 College of Business Administration, Chaohu University, Hefei, 238000 Anhui Province People’s Republic of China; 2 School of Computer Science and Engineering, Chongqing Three Gorges University, Chongqing, 404120 People’s Republic of China; 3 Faculty of Professional Finance and Accountancy, Shanghai Business School, Shanghai, 200235 People’s Republic of China; 4 School of Management, Shanghai University of Engineering Science, Shanghai, 201620 People’s Republic of China

**Keywords:** Complex networks, Nonlinear phenomena, Statistical physics

## Abstract

As a type of zero-determinant strategies, the extortion strategy was found to be an evolutionarily stable strategy in structural groups. However, instead of complex networks structure, this paper focus on a multi-group game in hypernetworks, using the framework of a gift giving game driven by replicator-like dynamics. We find that the extortion is evolutionarily stable in the hypernetwork structure. The extortion game in hypernetworks can promote the emergence of the cooperative behavior compared to the traditional dual-strategy game and the extortion game in complex networks. The results show that the cooperation behavior attracts most of the groups for the smaller benefit value. With the increase of benefit value, cooperators turn into defectors and extortioners, but cooperation behavior still survives in hypernetworks under extreme conditions. Moreover, small-scale groups are more conducive to cooperation.

## Introduction

Cooperation is a common and indispensable strategy observed in nature and social systems. The reason why selfish individuals sacrifice their own interests and choose cooperation is an evolutionary riddle, as it seemingly contradicts the fundamental Darwinian principles of natural selection. The evolutionary game theory provides a powerful framework to investigate the mechanisms behind the emergence of spontaneous cooperation among individuals engaged in repeated interactions. The evolutionary game theory has attracted considerable attention and been widely applied in various scientific studies^1–4^. There are many mechanisms that try to explain the emergence of cooperation^5–9^, such as reciprocity, kin selection, reputation, and networks. Press and Dyson proposed a novel class of strategies, referred to as the zero-determinant (ZD) strategies, which provided a new perspective for understanding the evolution of cooperation^10^. As a type of ZD strategies, the extortion strategy (${\mathrm{E}}_{\upchi }$) can unilaterally guarantee that one individual’s payoff is never smaller, but can be larger than the opponent’s payoff no matter what the strategy is chosen by opponents^10^. Parameter $\upchi$ is the extortion factor that determines how strongly ${\mathrm{ E}}_{\upchi }$ exploits cooperators. The extortion strategy can be viewed as the classic tit-for-tat strategy^11^ when $\upchi =1$. Extortioners use the conditional cooperative strategy to obtain more payoffs than their opponents. The experimental studies showed that human successfully extort a larger payoff from their opponents only when extortion strategy is part of Nash equilibrium^12^.

Zero-determinant strategies fundamentally changed the viewpoint on the Prisoner’s Dilemma^13^ and a large number of tracking studies were proposed. These studies show that the zero-determinant strategies have strong robustness in different conditions. Chen and Zinger^14^ found that the extortion strategy is extremely robust regardless of which evolutionary path is taken by the opponents. Hao et al.^15^ studied ZD strategies of the noisy repeated game and found that ZD strategies have high robustness to errors. However, extortion strategies are evolutionary unstable in a well-mixed population because extortioners gain nothing from their opponent when their opponents choose extortion or defection^16^. Hilbe and Nowak^17^ showed that an extortion strategy can be stable in a small population, and act as a catalyst for the emergence of cooperation in a homogeneously mixed population. To study the evolutionary stability of zero-determinant, the generous strategies were proposed by Steward and Plotkin^18^. In fact, both extortion and generous strategies enforce a linear relationship between the payoffs of his and his opponent. Extortionists who aim to outperform their opponents, but the payoff of generous players never exceed their opponents. The generous strategies can form a stable cluster and the local population cannot be invaded by other strategies. Generous strategies are robust to being replaced by other strategies in a well-mixed population.

The above studies assumed uniform interaction among individuals in the extortion game, i.e., all individuals in the population have game relationship with one another. The complex network theory is an effective tool to describe uncoupled game relationships. In a complex network, the nodes and edges are regarded as the players and game relationships among these players, respectively. Previous studies demonstrated that the network structure^19^, game payoffs measurement^20^, asymmetric cost^21^, compassion^22^ and emotions^23^ all play important roles in the evolution of cooperation. The game with extortion strategy in a structured population is systematically studied. Szolnoki and Perc^24,25^ considered that a stable extortion strategy can exist in structured populations and extortion strategy is the catalyst of unconditional cooperation. Mao et al.^26^ studied the roles of mutation mechanism for the evolution of cooperation with extortion strategies on clustered scale-free networks and found that small mutation rate can promote cooperation. Rong et al.^27^ investigated the game with generous strategies, extortion and unconditional defection strategies. The results showed that the proper aspiration level can promote the emergence of generous behaviors in a spatial prisoner’s dilemma (PD) game. Rong et al.^28^ studied the effect of the strategy-selection timescale on the evolution of extortion in the square lattices and scale-free networks. They observed that extortioners are able to build long-term stable relationships with cooperative neighbors, and the diversity of strategy-selection timescale further enhance the cooperative behavior. The scale-free networks may inhibit the maintenance of cooperation when the players’ payoffs are calculated as average payoffs, but the inhibiting effect disappears after the introduction of extortion strategies^29^.

**Figure 1:**
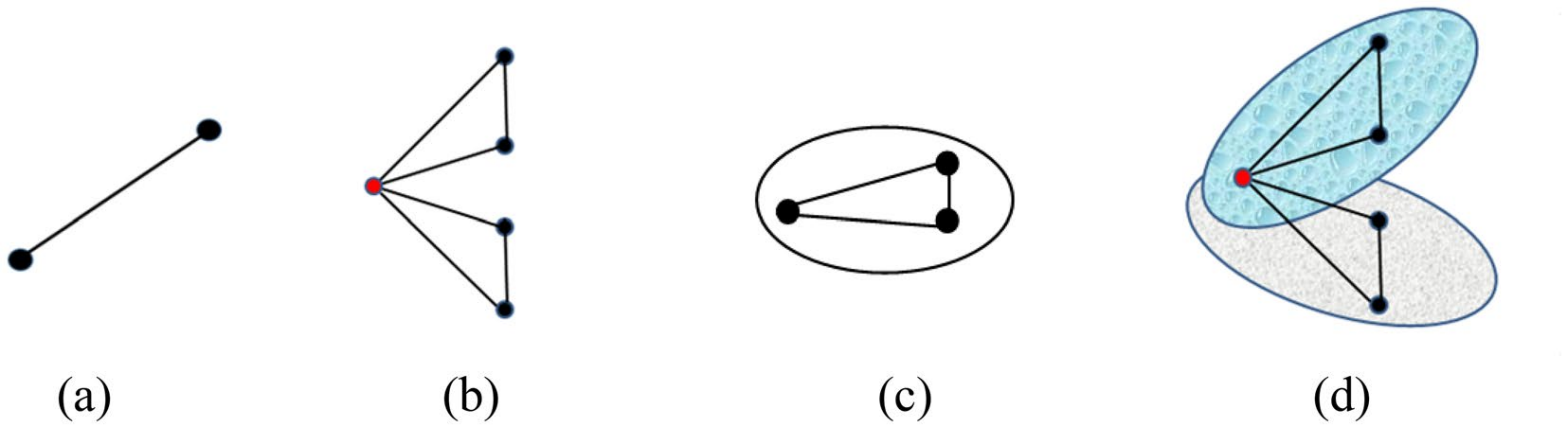
Different game relationships are expressed by network structure. Nodes and edges/hyperedges represent players and game relationships, respectively: (**a**) edge of complex network represents pairwise game relationship between two players; (**b**) degree of nodes in complex network show that player play a game with multiple opponents; (**c**) hyperedge shows that players participate in single-group game in hypernetwork and nodes in hyperedge are fully connected; (**d**) hyperdegree of nodes can divide the neighbors into different groups to show player participates in multiple-group game. The red node is the hub node or the overlapping node which can show the heterogeneous nature of realistic game relationship because it has more neighbors than other nodes.

The network game theory provides an effective analytical framework for studying the uncoupled game relationships among individuals^30,31^. In complex networks, the edge can express pairwise game relationships between two nodes (Fig. 1a). If a player engages in a game with multiple opponents, this can be characterized by the degree of nodes (Fig. 1b). However, the complex network structure cannot describe multiple-group game relationships in a social system. Recently, the network research moved to advanced complexity theory, i.e., hypernetworks, to better understand the multiple relationships between the components in real systems. In the hypergraph-based hypernetworks^32^, a hyperedge can contain any number of nodes and represent a single game group. The hyperdegree is the number of hyperedges of nodes, which can describe an individual play a game with multiple groups (Fig. 1c,d). By comparing Fig. 1b and d, we know that the hypernetwork structure in our model can better represent the heterogeneous nature of realistic multiple groups' game. Figure 1b expresses the hub nodes game with neighbors by complex networks which can't divide the neighbors into different groups. However, Fig. 1d can divide the neighbors into different groups by hyperedge and the overlapping node game with neighbors from different groups.

Therefore, we discuss what will happen when extortion strategies are introduced into a hypernetwork structure. In this paper, we try to address this question by exploring the evolution of extortion in the framework of a gift giving game driven by replicator-like dynamics. However, instead of focusing on the complex network structure, we emphasize the multiple-group game in the uniform scale-free hypernetworks. In "Hypernetwork model" section, we describe the algorithm used to generate the hypernetwork with a Poisson process. In "Game rules" section, we describe the game rule and strategy updating method reflecting the evolution of extortion on the static hypernetworks. In "Model simulation and result analysis" section, we simulate the game model to understand the evolutionary stability of extortion, and explore the influence of the model parameters on the game outcomes. The results show that extortion is evolutionary stable, but cooperation can still be maintained under extreme conditions. The size of groups and benefit value also significantly impact the evolutionary game on hypernetworks.

## Hypernetwork model

In this paper, we propose a uniform scale-free hypernetwork that is generated by a Poisson process and the evolution algorithm is as follows:

1. Initial condition: The hypernetwork starts from an initial seed of ${\text{m}}_{0}$ nodes, and a hyperedge contains these nodes.
2. Hypernetwork growth: A new batch of nodes joining the hypernetwork can be considered as an event. New node batches arrive at the hypernetwork according to a Poisson process, ${\text{N}}\left( {\text{t}} \right)$, with the rate $\uplambda$. At time ${\text{t}} = 1$, a new batch of ${\text{m}}_{1}$ nodes is added to the hypernetwork. These ${\text{m}}_{{1{ }}}$ new nodes and one old node are encircled by a new hyperedge, and a total of ${\text{m }}$ new hyperedges are constructed with no repetitive hyperedges at each time step.
3. Preferential attachment: The probability, $\prod \left( {{\text{k}}_{{{\text{iu}}}}^{{\text{h}}} \left( {\text{t}} \right)} \right)$, that the ${\text{m}}_{1}$ new nodes connect to the ${\text{uth}}$ old node of the ${\text{ith}}$ batch is proportional to the hyperdegree, ${\text{k}}_{{{\text{iu}}}}^{{\text{h}}} \left( {\text{t}} \right)$, such that
  1 $$\prod \left( {{\text{k}}_{{\text{u}}}^{{\text{h}}} } \right) = \frac{{{\text{k}}_{{\text{u}}}^{{\text{h}}} }}{{\mathop \sum \nolimits_{{\text{w}}} {\text{k}}_{{\text{w}}}^{{\text{h}}} }}$$

Our hypernetwork model can degenerate to the model in ref.^33^. In ref.^33^, new nodes arrive at hypernetworks with equal time intervals. However, in our model, new nodes arrive at hypernetworks according to Poisson process with the rate $\uplambda$. If the Poisson process rate $\uplambda = 1$, our hypernetwork model is equivalent to the model in ref.^33^.

**Figure 2:**
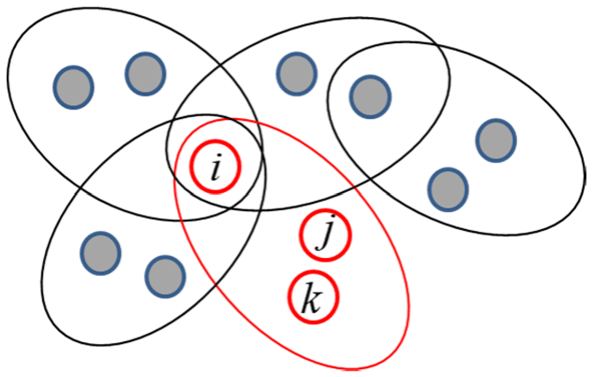
Multiple-group game relationships are represented by the uniform scale-free hypernetwork. The figure shows the scale of the hypernetwork N = 11 that is the total number of nodes. The node-degree of each hyperedge is equal to 3, which indicates that the hypernetwork is uniform and the nodes in each hyperedge can play a game with 2 opponents. Node i is the overlapping node with hyperdegree 4, and plays a game with ${ }2 \times 4$ opponents from four hyperedges or groups. In the same hyperedge, the node plays a game with each other. For example, in the red node and hyperedge, nodes are well-mixed and nodes ${\text{i}},{\text{j}}$, and ${\text{k }}$ play a game with each other.

**Figure 3:**
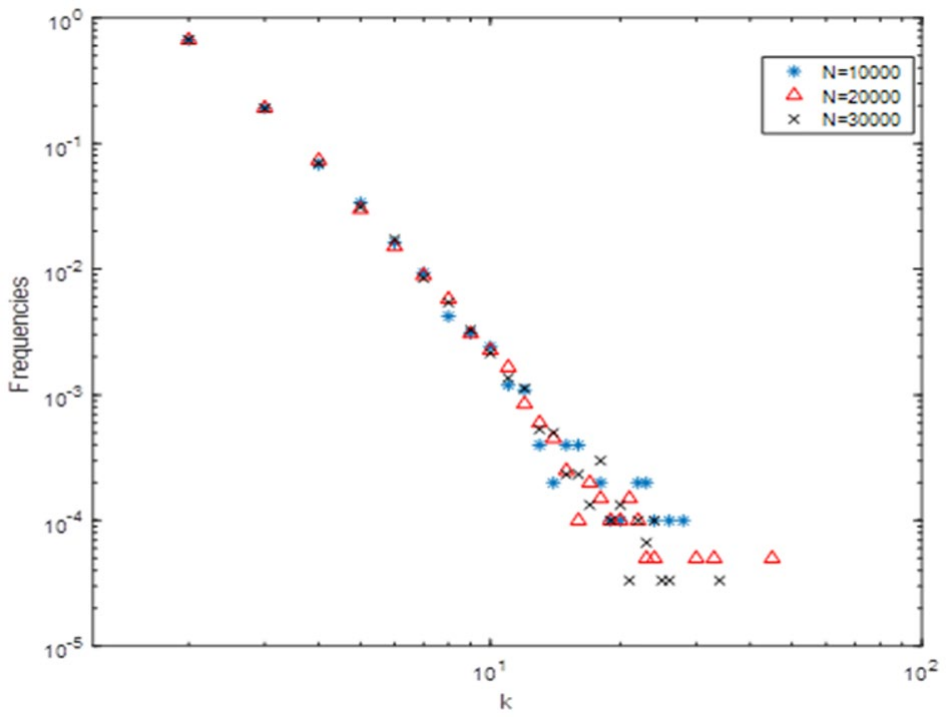
Hyperdegree distribution of nodes is shown in the double logarithmic coordinates. The horizontal coordinate is hyperdegree of nodes, and the vertical coordinate is the frequencies of hyperdegree of nodes occurrence. This figure indicates that nodes with few number of hyperdegree have high frequency, while nodes with more number of hyperdegree have low frequency. The results show that the hypernetwork structure in our model is a scale-free hypernetwork and has the property of the “Matthew effect”.

In hypernetworks, the circles represent hyperedges and the number of hyperedge of nodes is the hyperdergee of nodes. The number of nodes in hyperedge can be defined as the node-degree of hyperedges. We consider the extortion game in the uniform scale-free hypernetwork in which the number of nodes in each hyperedge is the same (Fig. 2) and the hyperdegree of nodes has the property of the “Matthew effect” (Fig. 3). In other words, the overlapping nodes play a game with a large number of groups, while the other nodes engage with a small number of groups.

Specifically, in our hypernetworks model, the number of new nodes is ${\text{m}}_{1} { }$ at each time step, so the number of nodes in each hyperedge is ${ }1 + {\text{m}}_{1}$ which means that each player plays with ${\text{m}}_{1}$ opponents in one hyperedge. If the hyperdegree of node ${\text{i }}$ is ${\text{k}}_{{\text{i}}}^{{\text{h}}}$, node ${\text{i }}$ plays extortion game with ${\text{k}}_{{\text{i}}}^{{\text{h}}} { }$ hyperedge, and the number of opponents is ${\text{k}}_{{\text{i}}}^{{\text{h}}} \cdot {\text{m}}_{1}$. The hyperdegree of node ${\text{k}}_{{\text{i}}}^{{\text{h}}}$ has the property of the “Matthew effect”, so each player plays with the different number of opponents which is determined by ${\text{k}}_{{\text{i}}}^{{\text{h}}}$. In the extortion game, players are located at the nodes of hypernetworks. The scale of hypernetworks, N, determines the total number of players. A hyperedge represents a game group in which the nodes are all connected and play a game with each other. The game relationships can be shown in Fig. 2.

In order to understand the characteristics of this hypernetworks structure, we simulate the model with different scales N according to the evolution algorithm above and the hyperdegree distribution of nodes is shown Fig. 3. It shows that the hyperdegree distribution of nodes is close to a straight line in the double logarithmic coordinates and is independent of scale N.

## Game rules

Each node can play a special prisoner's dilemma (PD) game, namely gift giving game, with its neighbors from different hyperedges. In the gift giving game, a cooperation node pays cost c to the neighbors who receive benefit b (b > c > 0). The players do not pay anything if they defect, and in that case their neighbors receive 0. Thus, the payoff of mutual cooperation is (b − c), and that of the mutual defection is 0. If a cooperator encounters a defector, they receive benefits − c and b, respectively. Where the parameter ${\text{b}}$ is the temptation to defect and can be called the benefit value. Taking extortioners with the extortion factor ${\chi }$ into consideration, each player can choose the game strategy from three strategies, including unconditional cooperation (C), unconditional defection (D), and extortion (${\text{E}}_{{\upchi }}$). Following closely Hilbe et al.^17^, the long-term payoff matrix involving the three strategies for node ${\text{i }}$ play a game with the node ${\text{j }}$ can be written as Table 1. From Table 1, we know that when a player's opponent chooses the extortion strategy, this player can get more payoffs by choosing the cooperation strategy. This suggests that cooperation is the best reply to extortion.

**Table 1:** Long-term payoff matrix of gift giving game involving extortion strategy.

	Node $j$		
	$C$	$D$	$E_{\chi }$
**Node** $i$			
$C$	$(b - c,b - c)$	$( - c,b)$	$\left( {\frac{{(b^{2} - c^{2} )}}{(b\chi + c)},\frac{{(b^{2} - c^{2} )\chi }}{(b\chi + c)}} \right)$
$D$	$(b, - c)$	$(0,0)$	$(0,0)$
$E_{\chi }$	$\left( {\frac{{(b^{2} - c^{2} )\chi }}{(b\chi + c)},\frac{{(b^{2} - c^{2} )}}{(b\chi + c)}} \right)$	$(0,0)$	$(0,0)$

In the evolutionary game, individuals play the game with neighbors from different groups (Fig. 2) and tend to adopt the neighbors’ strategies with high payoff. For game round ${\text{n}}$, the player ${\text{i }}$ obtains accumulated payoff ${\text{U}}_{{\text{i}}} \left( {\text{n}} \right){ }$ by playing gift giving game with the neighbors from different groups. The accumulated payoff of this round under pins the choice of strategy to be adopted by node ${\text{i }}$ in the next round. The accumulated payoff of node ${\text{i }}$ in game round ${\text{n}}$ can be expressed as,

2 $${\text{U}}_{{\text{i}}} \left( {\text{n}} \right) = \mathop \sum \limits_{{{\text{j}} = {\Omega }_{{\text{i}}} }} {\uppi }\left( {{\text{s}}_{{{\text{i}},{ }}} {\text{s}}_{{\text{j}}} } \right)$$

where $\Omega _{{\text{i}}}$ is the set of neighbors of node ${\text{i }}$ and ${\uppi }\left( {{\text{s}}_{{{\text{i}},{ }}} {\text{s}}_{{\text{j}}} } \right){ }$ is payoff in the strategy combination $\left( {{\text{s}}_{{{\text{i}},{ }}} {\text{s}}_{{\text{j}}} } \right)$.

As the game plays out, node ${\text{i }}$ calculates the accumulated payoff according to Eq. (2) and updates its behavior from the current round to the next. Node ${\text{i}}$ randomly selects a neighbor ${\text{j}}$ to compare the accumulated payoff. If the accumulated payoff of node i is greater than that of neighbor ${\text{j}}$, the strategy of node i does not change. If the accumulated payoff of node i is less than that of node ${\text{j}}$, the node ${\text{i }}$ will adjust its strategy from the current strategy ${\text{s}}_{{\text{i }}}$ to the strategy of node j (${\text{s}}_{{\text{j }}}$) with probability ${\text{P}}_{{\text{i}}}$. This behavior represents the replicator-like dynamics rule^34^. Probability ${\text{P}}_{{\text{i}}} { }$ can be expressed as,

3 $${\text{P}}_{{\text{i}}} \left( {{\text{s}}_{{\text{i}}} \leftarrow {\text{s}}_{{\text{j}}} } \right) = \frac{{{\text{U}}_{{\text{j}}} - {\text{U}}_{{\text{i}}} }}{{\left( {{\text{b}} + {\text{c}}} \right)\max \left( {{\text{k}}_{{\text{i}}}^{{\text{h}}} ,{\text{k}}_{{\text{j}}}^{{\text{h}}} } \right).{\text{m}}_{1} }}$$

where ${\text{U}}_{{\text{i}}}$ and ${\text{U}}_{{\text{j}}}$ are the accumulated payoffs of node ${\text{i }}$ and ${\text{j }}$ in the current game round, respectively. The denominator of Eq. (3) is to ensure that the probability ${\text{P}}_{{\text{i}}} \in \left[ {0,1} \right]$. Term ${ }\left( {{\text{b}} + {\text{c}}} \right)$ is the difference between the maximum and minimum of the payoff parameters shown in Table 1. Terms ${\text{k}}_{{\text{i}}}^{{\text{h }}}$ and ${\text{k}}_{{\text{j }}}^{{\text{h}}}$ represent the hyperdegree of nodes ${\text{i}}$ and ${\text{j}}$, respectively, and ${\text{max}}\left( {{\text{k}}_{{\text{i}}}^{{\text{h}}} ,{\text{k}}_{{\text{j}}}^{{\text{h}}} } \right).{\text{m}}_{1} { }$ is the total number of opponents of the player with the greater hyperdegree. Below we investigate how individual strategies evolve for different parameters in hypernetworks system.

## Model simulation and result analysis

We simulate a uniform hypernetwork model which is described above with 2000 nodes, and study the extortion game in hypernetworks by changing the relevant game variables. Suppose that at the game time ${\text{t}} \in \left( {{0, 1, 2, } \ldots } \right)$, the frequencies of the three evolutionary strategies are ${\text{f}}_{{\text{C}}} \left( {\text{t}} \right),{\text{f}}_{{\text{D}}} \left( {\text{t}} \right),\;{\text{and}}\;{\text{f}}_{{{\text{E}}_{{\upchi }} }} \left( {\text{t}} \right)$, respectively. In the initial state ${\text{t = 0}}$, individuals can randomly choose unconditional cooperation (C), unconditional defection (D), or extortion (${\text{E}}_{{\upchi }}$) as their initial strategy. The initial strategy satisfies that ${\text{f}}_{{\text{C}}} \left( 0 \right) = {\text{f}}_{{\text{D}}} \left( 0 \right) = {\text{f}}_{{{\text{E}}_{{\upchi }} }} \left( 0 \right)$. All players (nodes) obtain accumulated payoff by playing the gift giving game with their neighbors from different groups and update their strategies according to the replicator-like dynamics rule. The equilibrium frequencies of the strategies are obtained by averaging 3000 final generations after discarding 22,000 initial generations. A total 30 independent simulations are run to reduce the randomness.

### Stability of extortion evolution

**Figure 4:**
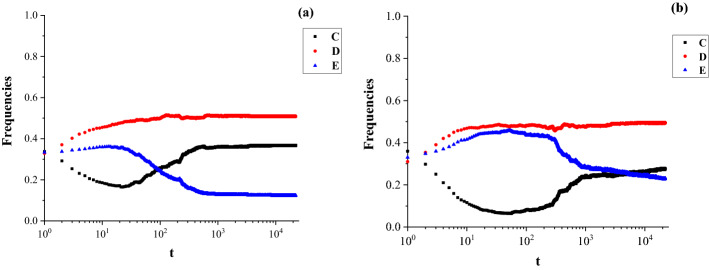
The extortion game in hypernetworks. We assume that the parameters ${\text{m}}_{1} = 2,{\text{b}} = 2$, and ${\text{c}} = 1$. Stability of cooperation (C), defection (D), and extortion (${\text{E}}_{{\upchi }}$) strategies: (**a**) relatively fair extortioner ($\upchi = 2$); (**b**) relatively unfair extortioner ($\upchi = 4$).

The extortion strategy is evolutionarily unstable in the well-mixed populations^16^, but it can coexist with other strategies and be stable in structured populations^17^. In order to study the stability of extortion strategies in hypernetworks. We simulate the evolution of the three strategies for different values of $\upchi$ and the results are shown in Fig. 4a,b.

Figure 4 shows that the extortion strategy coexists with cooperation (C) and defection (D), and became a long-term stable relationship with neighbors in hypernetworks. During the stable phase, the frequency of defection (D) is the highest (red line) and cooperation (C) (black line) occupies a large proportion of nodes. Initially, the three strategies have the same frequencies and players study their opponents’ strategies with the determined probability (Eq. 3) which depends on the game payoff of the previous game round. From Table 1, we know that the defection or extortion strategies can yield higher payoffs than the cooperation strategy. The cooperative individuals constantly learn the successful strategy according to Eq. (3).

For the relatively fair extortioner with ${\upchi } = 2$ (Fig. 4a), the cooperators learn strategies from their neighbors and turned into defectors to obtain higher payoff. The frequency of defection is stable at a high level in the early phase. As the game progressed, the extortion strategy changes to cooperation, which dramatically increases the frequency of cooperation and stabilizes the frequency of extortion at a low level. When encouraged by a higher extortion factor (${\upchi } = 4$), some cooperators become extortioners and defectors in the early phase of the game. Since a larger ${\chi }$ means that cooperators are more severely exploited by extortioners, the unfair extortioners force cooperators to adopt the exploitative behavior. However, the extortioners tend to make significant adjustments and develop into cooperators with the progress of the evolutionary game. The defection strategy has an absolute advantage and extortioners turn into cooperators as the game progressed. Figure 4a,b show that the positive feedback of the node pairs ${\text{C}} - {\text{E}}_{{\upchi }} { }$ are the important role in boosting and sustaining cooperation in hypernetworks, but the unfair extortioners reduce the frequency of cooperation and increase the frequency of extortion in the stable state.

In addition, the three strategies quickly reach a stable state, especially the defection strategy. This indicates that the game relationships represented by the hypernetwork structure can improve the speed of strategy learning for individuals. In the hypernetwork, individuals play a game with ${\text{k}}_{{\text{i}}}^{{\text{h}}} \cdot {\text{m}}_{1}$ opponents. For the network of the same size ${\text{N}}$, the hub nodes of hypernetworks have more opponents than that of complex networks. Once the hub nodes choose the defection strategy, their opponents learn to adopt extortion or defection which provides higher payoffs than cooperation. The resource advantages of hub nodes and the full connectivity of nodes in the same hyperedge accelerate the strategy propagation speed. In the process of strategies learning, individuals can adjust to their neighbors’ strategies, but the defection strategy lack room for adjustment and quickly settle into a stable state.

### Evolution of cooperation

**Figure 5:**
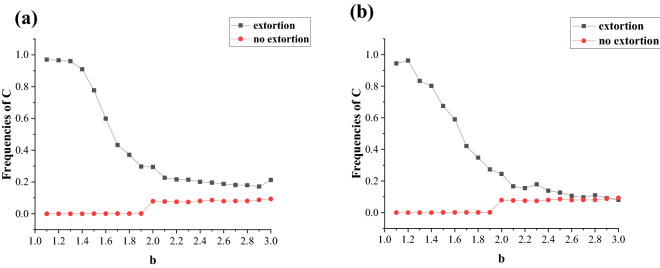
Comparison of cooperation evolution with and without extortion strategies for the different $\upchi$: (**a**) cooperation frequencies for $\upchi = 1.5$, (**b**) cooperation frequencies for ${{\upchi }} = 4$. In the simulations, the game parameters are ${\text{m}}_{1} = 3,\;{\text{and}}\;{\text{c}} = 1$.

In a traditional network game with only cooperation and defection strategies^35^, the heterogeneous scale-free networks and the accumulated payoff promote the emergence of cooperation. In the scale-free networks, the high-degree hubs have more neighbors than low-degree nodes, implying that high-degree hubs can obtain more payoffs than low-degree individuals. Once the hub nodes choose cooperative behavior, their neighbors also tend to choose the same behavior. The influence of the extortion strategy on the cooperation behavior in complex networks was discussed in the existing literature. Extortions can promote cooperation if the strategy updating is governed by the myopic best response^24,28^ or the replicator-like strategy updating rule^36,37^. Here we continue to investigate how the extortion strategy affects the evolution of cooperation by the replicator-like dynamics rule, but instead of the complex network structure, we focus on the game relationships represented by hypernetworks. We compare the frequencies of cooperation with and without extortion. The frequencies of cooperation for different $\upchi$ are shown in Fig. 5a,b.

Figure 5a,b show that the frequencies of cooperation in the hypernetwork depend on parameter ${\text{b }}$ for different values of ${{\upchi }}$. Compared with the evolution of extortion, the traditional dual-strategy version of the PD game lead to the disappearance of cooperation when the value of benefit factor is smaller than 2. As depicted in Fig. 5 (red lines), the frequency of cooperation tend to zero. In the dual-strategy game in hypernetworks, the hubs with more hyperedges interact with more neighbors and can find that choosing defection yield higher payoffs than cooperation. In the replicator dynamics rule of hypernetworks, the rational individuals with a low hyperdegree tend to study defection, and defection will bring about a mutual punishment situation. However, when b > 2 induce a small number of nodes may choose cooperation and the frequency of cooperation have a slight increase.

After adding the extortion strategy, the frequency of cooperation was improved significantly for the different parameters ${\text{b }}$ and ${{\upchi }}$, as illustrated in Fig. 5b (black lines). The results show that extortion provides an evolutionary “escape hatch” for cooperators to survive in the most adverse conditions (${\text{b}} = 3{ }$ and ${{\upchi }} = 4$). Although the introduction of extortioners will increase the number of those who exploit cooperators, the ${\text{C}} - {\text{E}}_{{{\chi }}}$ node pairs can achieve better results than ${\text{D}} - {\text{D}}$ or ${\text{D}} - {\text{E}}_{{\upchi }}$ node pairs. Therefore, the extortion strategy can act as catalysts for the dissemination of cooperative behavior in the evolutionary process, which leads to the dominance of cooperative behavior in the populations under steady state for small value of b. However, for large value of b, more nodes may choose defection strategy and ${\text{C}} - {\text{E}}_{{{\chi }}} { }$ node pairs are difficult to exist, which lead to the decreasing frequency of cooperation. In addition, compare to the extortion game in scale-free networks^37^, the cooperative behavior in hypernetworks can survive and further improve.

### The influence of benefit value b on the game

**Figure 6:**
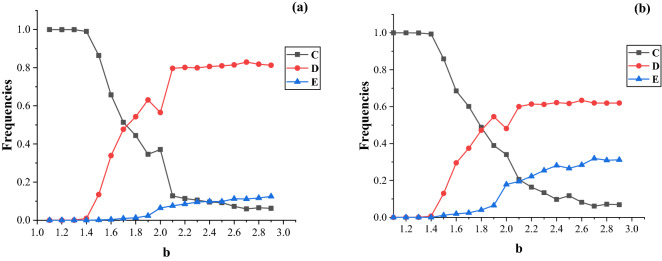
Frequencies of cooperation (C), defection (D), and extortion (E) as functions of benefit factor b for different value of extortion factor in steady state: (**a**) relatively fair extortioner (${\upchi } = 1.5$); (**b**) relatively unfair extortioner (${\upchi } = 3$.). The game parameters ${\text{b}} \in \left( {1,3} \right]$, ${\text{m}}_{1} = 2$, and ${\text{c}} = 1$.

The benefit value ${\text{b }}$ is the payoff obtained by defectors at no cost. In order to explore the influence of ${\text{b}}$ on the evolution of the three strategies in hypernetworks, we simulate our model and the frequencies of the three strategies for different ${\chi }$ are shown in Fig. 6.

We first study the frequencies of the three strategies as a function of the benefit factor ${\text{b }}$ in hypernetworks with a small extortion factor (${\upchi } = 1.5$). This factor entails extortioners inclined to share payoff with their neighbors fairly. Figure 6a indicates that the benefit value ${\text{b }}$ has significant influence on the frequencies of the three strategies. The frequency of the cooperation strategy decreases with ${\text{b}}$, whereas those of the extortion and defection strategies increases with ${\text{b}}$.

Figure 6a shows that when extortion evolves with cooperation and defection in hypernetworks, extortioners earn more but still offer positive payoff to their cooperative neighbors. With the help of extortion, the cooperation strategy dominates in the populations, but the defection and extortion strategies became extinct for ${\text{b}} \le 1.4$. This is similar to the results in Barabási-Albert scale-free networks, but it is different from the results based on the square lattice where a cooperation strategy rapidly became extinct for very small ${\text{b }}$^36^. In the evolution process of the game, the individuals learn the strategies of neighbors with high payoffs through the replicator dynamics rule. The overlapping nodes have more hyperedges and interact with more neighbors, thus they learn to choose defection or extortion to obtain higher accumulated payoff. If the overlapping nodes chose the defection or extortion strategy, the ${\text{D}} - {\text{D}}$ or ${\text{D}} - {\text{E}}_{{\upchi }} { }$ node pairs dominant the hyperedge, but the cooperative nodes still gain more accumulated payoffs from ${\text{C}} - {\text{E}}_{{\upchi }}$. It means that the positive feedback of ${\text{C}} - {\text{E}}_{{\upchi }}$ node pairs encourages more nodes to become cooperators for more accumulated payoff in the evolution process. Finally, cooperation flourished in hypernetworks in the stable state.

For the high values of b, such as ${\text{b}} > 1.4$, defection quickly spreads across the hypernetwork in the evolution process. As a result, defection occupy most nodes, the positive feedback of ${\text{C}} - {\text{E}}_{{\upchi }}$ disappears, which lead to the cooperation remain at a low frequency in the stable state. When ${\text{b }}$ is greater than 2.1, it start to have less influence on the frequencies of the three strategies. The results indicate that extortioners coexist with cooperators and defectors with high values of ${\text{b}}$ in hypernetworks. Compared to Fig. 6a,b, it can be concluded that the frequencies of the three strategies are similar for the different extortion factors, but the larger extortion factor ${\upchi } = 3{ }$ can increase the frequency of extortion and decrease that of defection.

### The influence of extortion factors ${{\varvec{\upchi}}}$ on the game

**Figure 7:**
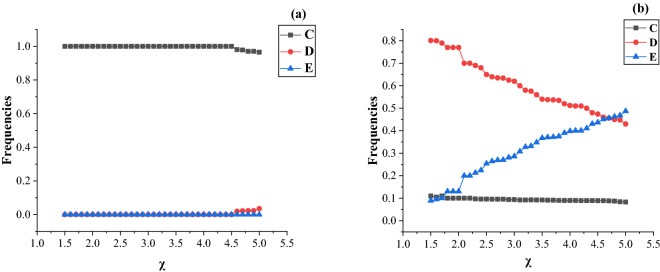
Frequencies of cooperation (C), defection (D), and extortion (E) as functions of extortion factor ${{\upchi }}$ for different value of benefit factor ${\text{b}} = 1.2$ and ${\text{b}} = 2.4$. In the simulations, the game parameters are ${\text{m}}_{1} = 2,{\text{and c}} = 1$.

Extortion factors determine how strongly extortioners exploit cooperators. We investigate the influence of the extortion factors ${ } {\upchi }$ on the game for the values of ${\text{b}} = 1.2{ }$ and ${\text{b}} = 2.4{ }$ in hypernetworks. The results are shown in Fig. 7. For the small ${\text{b}} = 1.2$, the extortion factor ${\chi }$ has slight influence on the frequencies of the three strategies and the cooperators dominate almost all nodes. The large extortion factor leads to a slight variation in cooperation and defection strategies. However, for the large ${\text{b}} = 2.4$, the extortion factor ${\chi }$ has greater influence on the frequencies of defection and extortion strategies. As the increases of the extortion factor ${\upchi }$, defectors are replaced by extortioner and the frequency of defection strategy is monotonically decreasing. It is worth mentioning that the frequency of cooperation is not affected by the extortion factor ${\upchi }$ and still survives in hypernetworks under extreme conditions with large ${{\upchi }}$ and ${\text{b}}$.

### Evolutionary games with different group sizes

**Figure 8:**
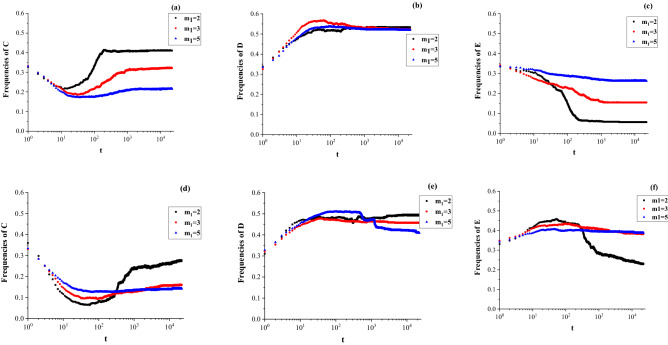
Evolutionary game with different group size ${\text{m}}_{1} = 2,3,{\text{and }}5$: (**a**)–(**c**) evolution of three strategies for ${\text{b}} = 2$ and ${{\upchi }} = 1.5$; (**d**)–(**e**) evolution of three strategies for ${\text{b}} = 2{ }$ and ${{\upchi }} = 3$.

The node degree of hyperedge is the number of nodes in each hyperedge, which represents the size of each game group in hypernetworks. In this paper, the hypernetwork is uniform, i.e., each hyperedge has the same number of nodes. The size of each game group is ${ }1 + {\text{m}}_{1}$. If individual ${\text{i }}$ plays the game with ${\text{k}}_{{\text{i}}}^{{\text{h}}}$ groups, it plays with ${\text{k}}_{{\text{i}}}^{{\text{h}}} \cdot {\text{m}}_{1}$ opponents. The group size is known to affect significantly the direct reciprocity and conditional cooperation in complex networks. In this paper, the node-degree of a hyperedge expresses the sizes of groups which can be adjusted by parameter ${\text{m}}_{1}$. Different value of $1 + {\text{m}}_{{1{ }}}$ are simulated and the evolution of the three strategies for different values of ${{\upchi }}$ are shown in Fig. 8.

Figure 8 shows that the different effects of the group size ${ }1 + {\text{m}}_{1}$ on the evolutionary game. As shown in Fig. 8a–c, the effects of group size on cooperation and extortion are stronger than that on defection for ${{\upchi }} = 1.5$. With the increase in ${\text{m}}_{1}$, the frequency of cooperation decreased, while that of extortion increased when the evolutionary game reach the stable state. When the group size ${\text{m}}_{1} { }$ increases, cooperators turn into extortioners and the majority of nodes adopt defection. For the larger extortion factor ${{\upchi }} = 3$, the influence of group size exhibits the same trend. The results show that small-scale groups are more conducive to the emergence of cooperation, while larger groups increase the frequency of extortion in the evolutionary game.

## Conclusions

As a type of zero-determinant strategies, the extortion strategy is evolutionary unstable in a well-mixed population^16^, but it was found to be an evolutionarily stable strategy in structural population when the game relationships are expressed by complex networks, such as scale free networks^26,27^ and regular lattice networks^36^. Games between groups exist widely in society. However, complex networks structure cannot describe the game relationships between multiple groups. The hyperedge of hypernetworks can divide opponents of players into different groups. In this paper, we study an evolutionary game with extortion strategy by using the scale free hypernetworks to represent the game relationships between groups. We show that extortion strategies are stable and improve the level of cooperation in hypernetworks. Moreover, the smaller benefit value ${\text{b }}$ and the small group sizes are more conducive to the emergence of cooperative behaviors.

How cooperation emerges and evolves are important research question. Game theory provides an effective theoretical framework to explore the emergence mechanism of spontaneous cooperation. The zero-determinant strategies elevate the understanding of evolutionary games and provide a new perspective for explaining the evolution of cooperation. The game in complex networks can help us to study non-uniform game relation in society. The early works about the extortion game in complex networks are of great significance for understanding cooperative behavior^26–29^ and decision-making mechanisms among individuals^38^. However, the study of evolutionary games in hypernetworks can better reveal the dynamic processes among groups. This paper provides effective theoretical framework for studying evolutionary games of multiple groups.

Now that game dynamics on hypernetworks are in the exploratory stage, many questions highlighted in this paper need to be further explored. Compared with complex networks, hypernetworks are more complex and derive many different statistical indicators, such as the overlapping nodes, the high clustering coefficient of nodes and the group size. These statistical indicators also have an important effect on behavior of groups. A possible path for future research is to study public goods game in hypernetworks. Complex networks structure can only represent the game relationship of single group. Public goods game is a typical game relationship with multiple groups. Hypernetworks can provide theoretical tools for public goods games among multiple groups and reveal the mechanism behind the emergence of cooperation among groups.

## Data Availability

The datasets used and/or analysed during the current study available from the corresponding author on reasonable request.
